# Draft Genome Sequence of the Rumen Methanogen *Methanobrevibacter olleyae* YLM1

**DOI:** 10.1128/genomeA.00232-16

**Published:** 2016-04-07

**Authors:** William J. Kelly, Dong Li, Suzanne C. Lambie, Faith Cox, Graeme T. Attwood, Eric Altermann, Sinead C. Leahy

**Affiliations:** AgResearch Limited, Grasslands Research Centre, Palmerston North, New Zealand

## Abstract

*Methanobrevibacter olleyae* YLM1 is a hydrogenotrophic methanogen, isolated from the rumen of a lamb. Its genome has been sequenced to provide information on the genomic diversity of rumen methanogens and support the development of approaches to reduce methane formation by ruminants.

## GENOME ANNOUNCEMENT

Members of the genus *Methanobrevibacter* are the dominant methanogens found in the digestive tract of ruminant livestock, and two different clades (*M. gottschalkii* and *M. ruminantium*) of closely related species constitute the bulk of the population (1–3). The *M. ruminantium* clade contains two described species, *M. ruminantium* (4) and *M. olleyae* (5). Here, we report the draft genome sequence of the hydrogenotrophic methanogen *M. olleyae* YLM1, which was isolated from the rumen of a lamb (6). 

The genome sequence of *M. olleyae* YLM1 was determined using pyrosequencing of 3-kb mate-paired libraries on a 454 GS FLX platform with titanium chemistry (Macrogen, South Korea) and combined with reads from an Illumina HiSeq 2000 platform (BGI, China), where a 2-kb mate-paired library was constructed with paired-end sequencing of 90-bp reads. Pyrosequencing reads were assembled using the Newbler assembler version 2.0 (Roche 454 Life Sciences, USA) and combined with Illumina reads using the SPAdes genome assembler version 3.0 (7). This resulted in 10 contigs in a single scaffold. Gap closure was managed using the Staden package (8), and gaps were closed using standard PCR techniques with Sanger sequencing, resulting in a single contig. One remaining gap, which is predicted to contain several tRNA genes, was unable to be closed. Protein-encoding genes were identified by Glimmer (9) and a GAMOLA/ARTEMIS (10, 11) software suite was used to manage genome annotation. Assignment of protein function to open reading frames was performed manually using results from BLASTp and the COG (Clusters of Orthologous Groups), Pfam, and TIGRFAM databases (12–14).

The draft genome sequence of *M. olleyae* YLM1 consists of a single 2,201,192-bp contig with a GC content of 26.9%, and 1,834 predicted protein-coding genes representing 75.7% of the genome. The YLM1 genome has a single 40-kb prophage and two CRISPR regions, but no plasmids. The overall gene content of *M. olleyae* YLM1 is largely comparable to that of *M. ruminantium* M1 (4), suggesting that the basic metabolism of these two hydrogenotrophic methanogens is similar. However, annotation of the YLM1 genome has highlighted two regions that potentially have important roles in cell functionality. The first of these is a 10-gene insertion containing the genes *hdr*ABC (CoB–CoM heterodisulfide reductase subunits), *com*ADE (enzymes involved in coenzyme M production), methanogenesis marker protein 16, an adhesin-like protein, and two hypothetical proteins. Consequently, YLM1 is predicted to be able to synthesize coenzyme M, which *M. ruminantium* M1 is unable to do (4). The second 9-gene region encodes a set of formate dehydrogenase genes, a hydrogenase maturation protein, an ATPase, and the *mrt*BDGA (methyl-coenzyme M reductase II) operon. The absence of *mrt* genes was noted in the M1 genome (4) and predicted to impact its ability to grow at differing hydrogen concentrations. Similar to M1, the YLM1 genome encodes 64 large adhesin-like proteins predicted to have a role in mediating interactions with other members of the rumen microbial community (15). Genomic information from *M. olleyae* YLM1 will complement genome sequences from other rumen methanogens (16).

### Nucleotide sequence accession number.

This whole-genome sequencing project has been deposited at DDBJ/EMBL/GenBank under the accession number CP014265.
